# CLR (C-Reactive Protein to Lymphocyte Ratio) Served as a Promising Predictive Biomarker for Cerebral Vasospasm in Aneurysmal Subarachnoid Hemorrhage (aSAH): A Retrospective Cohort Study

**DOI:** 10.3390/jcm13040940

**Published:** 2024-02-06

**Authors:** Ke Li, Dilaware Khan, Igor Fischer, Daniel Hänggi, Jan F. Cornelius, Sajjad Muhammad

**Affiliations:** 1 Department of Neurosurgery, Medical Faculty and University Hospital Düsseldorf, Heinrich-Heine-University Düsseldorf, 40225 Düsseldorf, Germany; 2 Department of Neurosurgery, King Edward Medical University, Lahore 54000, Pakistan; 3 Department of Neurosurgery, International Neuroscience Institute, 30625 Hannover, Germany; 4 Department of Neurosurgery, University of Helsinki, Helsinki University Hospital, 00290 Helsinki, Finland

**Keywords:** C-reactive protein to lymphocyte ratio, CLR, C-reactive protein, lymphocyte, NLR, PLR, MLR, mRS, cerebral vasospasm, delayed cerebral ischemia, chronic hydrocephalus, predictive value

## Abstract

**Background**: Subarachnoid hemorrhage is a devastating disease. Even after state-of-the-art treatment patients suffer from complications, including cerebral vasospasm (CVS), delayed cerebral ischemia (DCI), and chronic hydrocephalus (CH) following aneurysmal subarachnoid hemorrhage (aSAH). The aim of our study is to identify the predictive value of the C-reactive protein to lymphocyte ratio (CLR) for neurological functional outcome and complications after aSAH. **Methods**: We retrospectively analyzed a total of 166 aSAH patients who met the inclusion criteria enrolled in our study. Multivariate logistic regression analyses were performed to evaluate the independent risk factors. The predictive value of different models was compared by calculating the areas under the receiver operating characteristic (ROC) curve. **Results**: On-admission levels of CLR in patients with poor outcomes (6 months mRS 3–6), CVS, DCI, and CH were significantly higher than those in patients with good outcomes (6 months mRS 0–2), non-CVS, non-DCI, and non-CH. Multivariate logistic regression analysis revealed that admission CLR was independently associated with CVS (OR [95% CI] 2.116 [1.507–2.971]; *p* < 0.001), and DCI (OR [95% CI] 1.594 [1.220–2.084]; *p* = 0.001). In ROC analysis, the area under the curve (AUC) of CLR for poor outcomes (6 months mRS 3–6), CVS, DCI, and CH prediction were (AUC [95% CI] 0.639 [0.555–0.724]; *p* = 0.002), (AUC [95% CI] 0.834 [0.767–0.901]; *p* < 0.001), (AUC [95% CI] 0.679 [0.581–0.777]; *p* < 0.001), and (AUC [95% CI] 0.628 [0.543–0.713]; *p* = 0.005) revealing that admission CLR had a favorable predictive value for CVS after aSAH. The sensitivity and specificity of admission CLR for CVS prediction were 77.1% and 75.4%. On-admission CLR of 0.757 mg × 10^−6^ was identified as the best cutoff threshold to discriminate between CVS and non-CVS (CVS: CLR < 0.757 mg × 10^−6^ 11/100 [11.0%] vs. CLR ≥ 0.757 mg × 10^−6^ 37/66 [56.1%]; *p* < 0.001). **Conclusions**: High levels of on-admission CLR serve as an independent risk factor for CVS and DCI after aSAH. Admission CLR is an easy-to-quantify laboratory parameter that efficiently predicts the CVS after aSAH, which can provide some guidance for clinicians to evaluate for possible progression and treatment strategies in patients with aSAH.

## 1. Introduction

Subarachnoid hemorrhage (SAH) is a serious stroke subtype that is mainly caused by ruptured aneurysms and has high mortality and disability [1]. Around 85% of aneurysmal SAH (aSAH) emerges through the rupture of intracranial aneurysms [2]. The incidence of aSAH was reported as 9/100,000 people per year in the USA, and 600,000 cases per year globally [2]. The premature mortality rate of aSAH was approximately 40% and patients in almost 10–20% of cases died before acquiring medical attention or during transportation [3]. Cerebral vasospasm (CVS), delayed cerebral ischemia (DCI), and chronic hydrocephalus (CH) are life-threatening complications in patients with aSAH [4]. CVS has been defined as the narrowing of large cerebral arteries usually occurring 3–14 d after SAH in almost 70% of patients [5]. CVS is one of the major causes of morbidity and mortality after aSAH in patients surviving from initial aneurysmal rupture. CVS can be diagnosed through transcranial Doppler ultrasonography (TCD), digital subtraction angiography (DSA), or computed tomography angiography (CTA)/perfusion (CTP) [6]. For survivors of aSAH, DCI serves as an important modifiable prognostic factor and occurs mostly on the fourth day or later. CVS was usually considered to be the cause of DCI [7]. However, cortical spreading ischemia, microcirculatory constriction, and/or thrombosis have also been shown to contribute to DCI formation [3]. Furthermore, hitherto, the etiology of the occurrence of CH after aSAH is still unclear. The diagnosis of CH depends on radiographic enlargement of the ventricles in the absence of clinical symptoms and signs of dramatically increasing intracranial pressure [8]. Ventriculoperitoneal shunting is the most common method of placing a permanent shunt catheter for patients requiring permanent CSF diversion [9]. CVS, DCI, and CH cause high morbidity and mortality after aSAH. An accurate prediction of clinical functional outcome and complications is essential to the possible progression and the choices of treatment strategies.

A previous study stated that high levels of neutrophil to lymphocyte ratio (NLR) might serve as an independent predictive factor associated with an unfavorable functional outcome after aSAH [10]. Platelet to lymphocyte ratio (PLR) was reported to weakly associate with the mortality in hospital after aSAH [11]. Little research focused on the correlation between monocyte to lymphocyte ratio (MLR) and the outcome after aSAH. Furthermore, elevated admission serum C-reactive protein (CRP) levels were correlated with poor outcome in patients with aSAH [12]. Similarly, a high count of platelets in patients with aSAH was associated with the elevated occurrence of DCI after aSAH [13]. Recently, Zhang et al. employed a novel biomarker named C-reactive protein to lymphocyte ratio (CLR) and demonstrated that admission CLR value serves as a feasible biomarker to predict the clinical prognosis of patients with aSAH [14]. However, the predictive value of the admission CLR value for neurological complications including CVS, DCI, and CH is still not clear. Hence, the purpose of the present study is to identify the predictive value of admission CLR, NLR, PLR, and MLR values in neurological functional outcome and complications after aSAH.

## 2. Methods

### 2.1. Study Design

**Figure 1:**
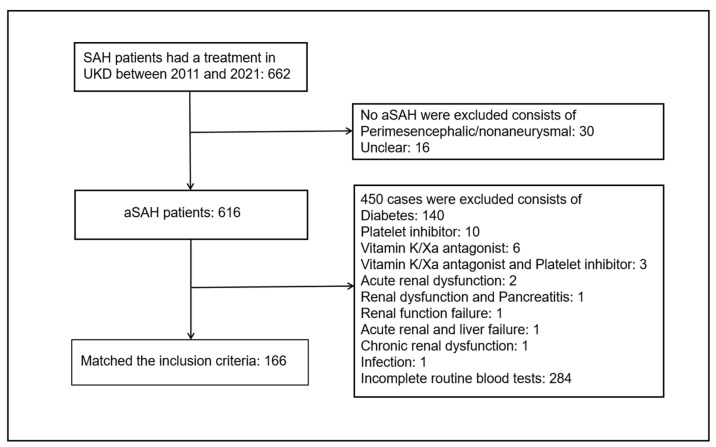
Flowchart of patient enrollment.

We retrospectively analyzed 662 patients with SAH, who had been hospitalized within 48 h and underwent laboratory examination in the Department of Neurosurgery of the Medical Faculty and University Hospital Düsseldorf from January 2011 to October 2021 and were diagnosed with a computed tomography (CT) scan. The clinical data including patient demographics (e.g., age, sex) and medical history (e.g., hypertension, diabetes, etc.) from these patients were collected via medical records and/or outpatient follow-up. The inclusion criteria for enrollment were as follows: (1) age above 18 years, (2) hospitalized within 48 h in the emergency unit, (3) detected SAH by CT scan, and the diagnosis of the aSAH was achieved by CTA or DSA. The study exclusion criteria were as follows: (1) no aneurysms detected by CTA or DSA, (2) patients without complete clinical data and laboratory examination, (3) having complications with infection, immune dysfunction, blood system diseases, organ function damage, or major infectious diseases, which had significantly influenced the laboratory examination. Forty-six patients were excluded because in them SAH was not caused by aSAH, consisting of 30 cases with perimesencephalic/nonaneurysmal and 16 cases that were unclear. Furthermore, 140 patients were diagnosed with diabetes, 10 cases took the platelet inhibitor, six cases had the vitamin K/Xa antagonist, three cases took the vitamin K/Xa antagonist and platelet inhibitor together, two cases were diagnosed with acute renal dysfunction, one case with renal dysfunction and pancreatitis, one case with renal function failure, one case with acute renal and liver failure, one case with chronic renal dysfunction, and one case with infection that could have significantly influenced the peripheral serum biomarkers; 284 cases with no data on the admission lymphocyte count were excluded. Finally, 166 patients aged above 18 years (ranging from 21.00 to 84.00 years) met the inclusion criteria and were enrolled in our study (Figure 1). The strict inclusion/exclusion criteria and the use of standardized data collection procedures minimized the impact of selection bias and information bias. All 166 patients had complete data. The study was reported in accordance with the STROBE (Strengthening the Reporting of Observational Studies in Epidemiology) reporting guidelines.

### 2.2. Clinical Data Collection and Neurological Complication Evaluation

We followed our standardized diagnostic and treatment charts as previously described [15]. SAH was detected by a CT scan. CTA and DSA were employed for further evaluation of neuroradiological data such as modified Fisher (mFisher) Grading Scale, intracerebral hemorrhage, subdural hemorrhage, and aneurysmal locations and sizes. The admission status was measured by the World Federation of Neurosurgical Societies (WFNS) grade, Glasgow Coma Score (GCS). The treatment methods (coiling or clipping) were dependent on an interdisciplinary approach. Our treatment protocol at the neurological intensive care unit (NICU) included hourly neurological monitoring, continuous invasive blood pressure and body temperature measurements, daily TCD, and the application of nimodipine for 21 days starting from the day of admission. CTA and CTP were performed for confirmation upon suspicion of CVS. The diagnostic criteria for CVS included symptomatic vasospasm (secondary clinical decline with new neurological deficits) or radiological vasospasm (diagnosed with digital subtraction angiography, with transcranial Doppler (TCD) and with CT perfusion (CTP) studies). All patients underwent daily TCD and CT perfusion on day 1, 4, 7, 10, and 14 on a routine basis. On any suspicion of CVS, the patients received DSA to confirm CVS and to treat CVS with intraarterial nimodipine. Symptomatic vasospasm was defined as the development of new focal neurological signs, deterioration in level of consciousness, or both, when the cause was felt to be ischemia attributable to vasospasm after other possible causes of worsening (for example, hydrocephalus, seizures, metabolic derangement, infection, or oversedation) had been excluded. Angiographic vasospasm was defined as moderate-to-severe arterial narrowing on DSA not attributable to atherosclerosis, catheter-induced spasm, or vessel hypoplasia, as determined by a neuroradiologist. TCD vasospasm was defined as a mean flow velocity in any vessel >120 cm/s. CTP vasospasm diagnosed with the patients showing significant perfusion deficits with a mean transient time ≥5 s in CTP and DSA excluded the perfusion deficits caused by occlusion of the blood vessels (such as by clots) with/without a new focal neurological deficit within 3 days to 2 weeks after SAH [12,15,16]. Furthermore, as previously mentioned, DCI was defined as combining the presence of cerebral infarction on a CT or MR scan of the brain within 6 weeks after SAH, or the latest CT or magnetic resonance imaging (MRI) scan made before death within 6 weeks, or proven at autopsy, not present on the CT or MRI scan between 24 and 48 h after early aneurysm occlusion, and not attributable to other causes such as surgical clipping or endovascular treatment with the occurrence of focal neurological impairment (such as hemiparesis, aphasia, apraxia, hemianopia, or neglect), or a decrease of at least two points on the Glasgow Coma Scale (either on the total score or on one of its components [eye, motor on either side, verbal]). This should last for at least 1 h, should not be apparent immediately after aneurysm occlusion and cannot be attributed to other causes employing clinical assessment, CT or MRI scanning of the brain, and appropriate laboratory studies [17]. Hydrocephalus was diagnosed by the following standards: (1) Evans index > 0.3 (the ratio of the greatest distance between bilateral anterior horns of the lateral ventricles and the greatest internal distance of the skull); and (2) enlargement of the anterior horns of the lateral ventricles, temporal horns, and the third ventricle accompanied with periventricular cerebral edema [18]. CH was defined by hydrocephalus that occurred postoperatively for 2 weeks. Seizures were mainly diagnosed by clinical symptoms, signs, and outpatient follow-up. Neurological functional outcome was measured by modified Rankin Scale (mRS) after admission at 6 months collected from outpatients at follow-up. Functional outcome was dichotomized into good (mRS Score 0–2) and poor (mRS Score 3–6) [10].

### 2.3. Admission Serum Biomarker Collection

The quantitative variables including the admission WBC, platelet, neutrophil, lymphocyte, and monocyte count, and the levels of CRP were obtained from routine blood tests and comprehensive biochemical tests within 24 h at the hospital emergency unit. Calculations of CLR, NLR, PLR, and MLR used the ratio by the level of CRP, and the neutrophil, platelet, and monocyte count dividing the lymphocyte count.

### 2.4. Statistical Analysis

All statistical analyses were performed using SPSS, version 25.0 (IBM Corp., Armonk, New York, NY, USA). We employed the Kolmogorov–Smirnov test to test the normality of the variables. The normally distributed variables were defined as the mean ± standard deviation (SD) and the non-normally distributed variables as the median and interquartile range (IQR). The categorical variables were defined as numbers and percentages. Univariate analysis was performed by dividing the cohort into two groups according to the dependent variables. We employed the independent Student t-test and Mann–Whitney U test to compare the differences between the two groups of normally distributed and non-normally distributed variables, respectively. Differences in the categorical variables were compared using the Chi-square test or Fisher exact test. The associations between the risk factors and CVS and DCI were performed using multivariate logistic regression models, which were presented by the calculations of the odds ratio (OR) and 95% confidence interval (CI). Only statistically significant variables with a *p* < 0.005 in univariate analysis were included in the multivariate logistic regression. The independent risk factors were evaluated by multivariate logistic regression [19]. The receiver operating characteristic (ROC) curves were used to evaluate the predictive value [11]. *p* < 0.05 was considered to be a statistically significant difference.

## 3. Results

### 3.1. Baseline Characteristics

The baseline characteristics of 166 aSAH patients are shown in Table 1. The mean age of the cohort was 55.58 (SD, 12.22) years ranging from 21.00 to 84.00 years. A total of 114 (68.7%) patients were females. A total of 117 (70.5%) patients were diagnosed with hypertension. The median admission WFNS grade was 2 (IQR, 1–5) and the median GCS score was 13 (IQR, 4–15). Additionally, the median of CRP, WBC, platelet, neutrophil, lymphocyte, monocyte, CLR, NLR, PLR, and MLR in the cohort were 0.50 (IQR, 0.20–1.40) mg/dL, 13.35 (IQR, 10.58–15.90) × 10^9^/L, 230.50 (IQR, 196.75–278.25) × 10^9^/L, 11.17 (IQR, 8.52–14.00) × 10^9^/L, 1.05 (IQR, 0.74–1.60) × 10^9^/L, 0.78 (IQR, 0.50–1.00) × 10^9^/L, 0.50 (IQR, 0.17–1.56) mg × 10^−6^, 11.00 (IQR, 6.24–16.74), 218.87 (IQR, 143.57–323.11), and 0.63 (IQR, 0.43–0.90), respectively. Furthermore, neuroradiological data in our study included on-admission mFisher scores, intracerebral and subdural hemorrhage status, and aneurysmal locations and sizes. The median of the mFisher score was 4 (IQR, 3–4). There were 37 (22.3%) aSAH patients with intracerebral hemorrhage and 14 (8.4%) patients with subdural hemorrhage. The locations of aneurysms displayed different distributions: 77 (46.4%) cases with anterior cerebral arteries (ACA) and/or anterior communicating artery (ACOM), 38 (22.9%) cases with middle cerebral artery (MCA), 19 (11.4%) cases with posterior communicating artery (PCOM), and nine (5.4%) and 23 (13.9%) cases with internal carotid artery (ICA) and posterior circulation (PC), respectively. The sizes of aneurysms also showed different distributions: 49 (29.5%) cases with 0–4.9 mm, 46 (27.7%) cases with 5–6.9 mm, 21 (12.7%) cases with 7–9.9 mm, 19 (11.4%) cases with 10–19.9 mm, and six (3.6%) cases with ≥20 mm. The data of 25 (15.1%) cases were missing. Endovascular coiling was performed in 46 (27.7%) cases and surgical clipping was performed in 114 (68.7%) cases. Six (3.6%) cases had no treatment. There were 48 (28.9%) aSAH patients diagnosed with CVS, 53 (31.9%) cases with DCI, 88 (53.0%) cases with CH, and 23 (13.9%) cases with seizures in the cohort, respectively. The median of 6 months mRS (represented the neurological functional outcome) was 3 (IQR, 1–5).

### 3.2. Univariate Analysis on CVS, mRS (Good 0–2 vs. Poor 3–6), DCI, and CH after aSAH

As shown in Table 2, the included 166 aSAH patients were categorized by CVS. Univariate analysis revealed that the on-admission serum levels of CRP (median [IQR] CRP 1.95 [0.80–3.10] mg/dL vs. 0.30 [0.18–0.80] mg/dL; *p* < 0.001), WBC (median [IQR] WBC 14.05 [11.68–18.33] × 10^9^/L vs. 12.60 [10.10–15.70] × 10^9^/L; *p* = 0.040), neutrophil (median [IQR] neutrophil 11.95 [10.05–15.95] × 10^9^/L vs. 10.49 [7.88–13.49] × 10^9^/L; *p* = 0.007), and CLR (median [IQR] CLR 1.83 [0.77–4.46] mg × 10^−6^ vs. 0.30 [0.12–0.76] mg × 10^−6^; *p* < 0.001), NLR (median [IQR] NLR 12.90 [9.80–20.59] vs. 8.74 [5.54–15.97]; *p* = 0.001), PLR (median [IQR] PLR 262.08 [189.38–397.88] vs. 215.88 [135.60–294.97]; *p* = 0.012), and MLR (median [IQR] MLR 0.74 [0.59–1.11] vs. 0.58 [0.39–0.77]; *p* = 0.001) in patients with CVS were significantly higher than those in patients without CVS. The admission lymphocyte count (median [IQR] lymphocyte 0.93 [0.60–1.32] × 10^9^/L vs. 1.17 [0.80–1.62] × 10^9^/L; *p* = 0.010) in patients with CVS was significantly less than that in patients without CVS. The patients without CVS had favorable admission status, meaning a lower WFNS grade (median [IQR] WFNS grade 2 [1,2,3,4,5] vs. 3 [2,3,4,5]; *p* = 0.018) and higher GCS score (median [IQR] GCS score 14 [5,6,7,8,9,10,11,12,13,14,15] vs. 11 [3,4,5,6,7,8,9,10,11,12,13,14]; *p* = 0.016) than those in patients with CVS. The patients without CVS had better neurological functional outcome (median [IQR] 6 months mRS 2 [1,2,3,4,5] vs. 5 [2,3,4,5,6]; *p* < 0.001) and less incidence of neurological complications including DCI (number [%] DCI 26 [22.0%] vs. 27 [56.3%]; *p* < 0.001), CH (number [%] CH 53 [44.9%] vs. 35 [72.9%]; *p* = 0.001), and seizures (number [%] seizures 12 [10.2%] vs. 11 [22.9%]; *p* = 0.031) than those in patients with CVS. Similarly, admission serum levels of CLR in patients with poor outcome (6 months mRS 3–6) (median [IQR] CLR 0.77 [0.20–2.74] mg × 10^−6^ vs. 0.34 [0.13–0.83] mg × 10^−6^; *p* = 0.002; Supplemental Table S1), DCI (median [IQR] CLR 1.34 [0.21–3.60] mg × 10^−6^ vs. 0.43 [0.13–0.84] mg × 10^−6^; *p* < 0.001; Supplemental Table S2), and CH (median [IQR] CLR 0.75 [0.21–1.98] mg × 10^−6^ vs. 0.33 [0.13–0.95] mg × 10^−6^; *p* = 0.004; Supplemental Table S3) were significantly higher than those in patients with good outcome (6 months mRS 0–2), non-DCI, and non-CH. Admission serum levels of NLR in patients with poor outcome (6 months mRS 3–6) (median [IQR] NLR 12.75 [7.11–19.17] vs. 8.74 [5.32–13.32]; *p* = 0.001; Supplemental Table S1), DCI (median [IQR] NLR 12.76 [8.14–22.39] vs. 9.33 [5.90–15.85]; *p* = 0.012; Supplemental Table S2), and CH (median [IQR] NLR 12.90 [8.63–19.17] vs. 8.46 [4.83–13.05]; *p* < 0.001; Supplemental Table S3) were significantly higher than those in patients with good outcome (6 months mRS 0–2), non-DCI, and non-CH. Admission serum levels of MLR in patients with poor outcome (6 months mRS 3–6) (median [IQR] MLR 0.70 [0.51–1.12] vs. 0.55 [0.39–0.74]; *p* = 0.001; Supplemental Table S1), DCI (median [IQR] MLR 0.69 [0.56–1.32] vs. 0.58 [0.42–0.81]; *p* = 0.010; Supplemental Table S2), and CH (median [IQR] MLR 0.69 [0.47–1.08] vs. 0.56 [0.39–0.74]; *p* = 0.007; Supplemental Table S3) were significantly higher than those in patients with good outcome (6 months mRS 0–2), non-DCI, and non-CH. Admission serum levels of PLR in patients with CH (median [IQR] PLR 240.17 [163.58–352.87] vs. 198.43 [132.50–296.56]; *p* = 0.041; Supplemental Table S3) were significantly higher than those in patients with non-CH.

### 3.3. Multivariate Logistic Analysis of Risk Factors for Poor Outcome (6 Months mRS 3–6), CVS, DCI, and CH

Due to admission CLR being dependent on the levels of CRP, we only included the CLR in the multivariate logistic analysis. Neurological functional outcome and complications were excluded in the multivariate logistic analysis of variables associated with CVS, DCI, and CH. As shown in Table 3, multivariate logistic regression analysis revealed that the on-admission CLR was independently associated with CVS (OR [95% CI] 2.116 [1.507–2.971]; *p* < 0.001). However, NLR (OR [95% CI] 1.007 [0.946–1.072]; *p* = 0.830), and MLR (OR [95% CI] 0.843 [0.372–1.909]; *p* = 0.682) were not independently associated with the occurrence of CVS. Similarly, multivariate logistic regression analysis demonstrated that on-admission CLR was independently associated with DCI (OR [95% CI] 1.594 [1.220–2.084]; *p* = 0.001; Supplemental Table S5) after aSAH. However, high levels of on-admission CLR were not an independent risk factor for poor outcome (6 months mRS 3–6) (OR [95% CI] 1.474 [0.997–2.177]; *p* = 0.052; Supplemental Table S4) and CH (OR [95% CI] 0.957 [0.843–1.087]; *p* = 0.501; Supplemental Table S6) after aSAH.

### 3.4. Predictive Value of CLR for CVS, DCI, Poor Outcome (6 Months mRS 3–6), and CH after aSAH

**Figure 2:**
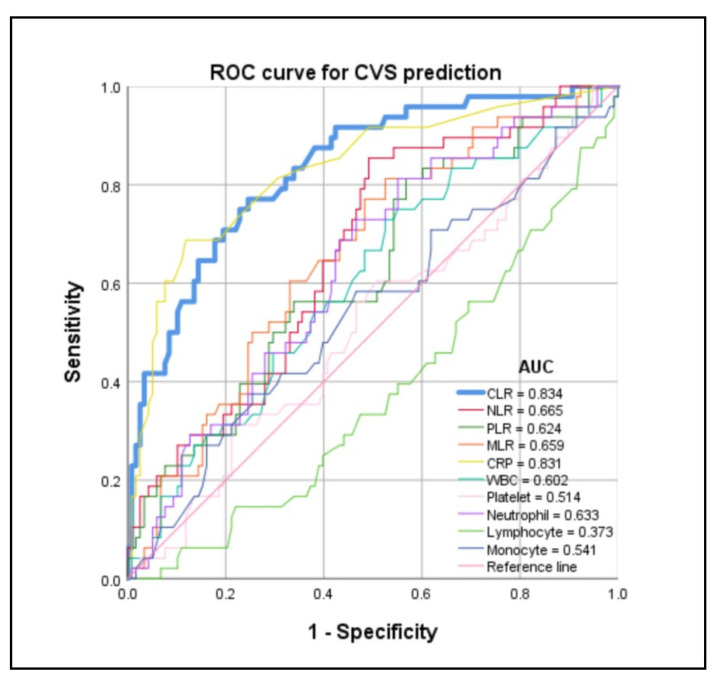
Predictive value of each admission serum biomarkers for the cerebral vasospasm (CVS) using receiver operating characteristics (ROC) curve.

The ROC curves were performed to clarify the predictive value of admission CLR, NLR, PLR, MLR, CRP, WBC, platelet, neutrophil, lymphocyte, and monocyte in CVS, DCI, CH, and poor outcome (6 months mRS 3–6) after aSAH. The area under the curve (AUC) of admission CLR for the predicted of CVS, poor outcome (6 months mRS 3–6), DCI, and CH were (AUC [95% CI] 0.834 [0.767–0.901]; *p* < 0.001; Figure 2; Table 4), (AUC [95% CI] 0.639 [0.555–0.724]; *p* = 0.002; Supplemental Figure S1; Supplemental Table S7), (AUC [95% CI] 0.679 [0.581–0.777]; *p* < 0.001; Supplemental Figure S2; Supplemental Table S8), and (AUC [95% CI] 0.628 [0.543–0.713]; *p* = 0.005; Supplemental Figure S3; Supplemental Table S9), respectively. The AUC of admission NLR for the predicted of CVS, poor outcome (6 months mRS 3–6), DCI, and CH were (AUC [95% CI] 0.665 [0.577–0.752]; *p* = 0.001; Figure 2; Table 4), (AUC [95% CI] 0.644 [0.560–0.728]; *p* = 0.001; Supplemental Figure S1; Supplemental Table S7), (AUC [95% CI] 0.621 [0.528–0.714]; *p* = 0.012; Supplemental Figure S2; Supplemental Table S8), and (AUC [95% CI] 0.681 [0.601–0.762]; *p* < 0.001; Supplemental Figure S3; Supplemental Table S9), respectively. The AUC of admission PLR for the predicted of CVS, poor outcome (6 months mRS 3–6), DCI, and CH were (AUC [95% CI] 0.624 [0.531–0.718]; *p* = 0.012; Figure 2; Table 4), (AUC [95% CI] 0.582 [0.495–0.668]; *p* = 0.069; Supplemental Figure S1; Supplemental Table S7), (AUC [95% CI] 0.545 [0.448–0.642]; *p* = 0.350; Supplemental Figure S2; Supplemental Table S8), and (AUC [95% CI] 0.592 [0.505–0.679]; *p* = 0.041; Supplemental Figure S3; Supplemental Table S9), respectively. The AUC of admission MLR for the predicted of CVS, poor outcome (6 months mRS 3–6), DCI, and CH were (AUC [95% CI] 0.659 [0.571–0.748]; *p* = 0.001; Figure 2; Table 4), (AUC [95% CI] 0.654 [0.571–0.737]; *p* = 0.001; Supplemental Figure S1; Supplemental Table S7), (AUC [95% CI] 0.624 [0.533–0.715]; *p* = 0.010; Supplemental Figure S2; Supplemental Table S8), and (AUC [95% CI] 0.622 [0.537–0.707]; *p* = 0.007; Supplemental Figure S3; Supplemental Table S9), respectively. The results revealed that admission CLR had a favorable predictive value for CVS after aSAH. The sensitivity and specificity of admission CLR for CVS prediction were 77.1% and 75.4% (Table 4). An admission CLR of 0.757 mg × 10^−6^ was identified as the best cutoff threshold to discriminate between CVS and non-CVS (CVS: CLR < 0.757 mg × 10^−6^ 11/100 [11.0%] vs. CLR ≥ 0.757 mg × 10^−6^ 37/66 [56.1%]; *p* < 0.001) (Table 5). The incidence of DCI (DCI: CLR < 0.757 mg × 10^−6^ 20/100 [20.0%] vs. CLR ≥ 0.757 mg × 10^−6^ 33/66 [50.0%]; *p* < 0.001) and CH (CH: CLR < 0.757 mg × 10^−6^ 45/100 [45.0%] vs. CLR ≥ 0.757 mg × 10^−6^ 43/66 [65.2%]; *p* = 0.011) in patients with CLR < 0.757 mg × 10^−6^ were significantly lower than those in patients with CLR ≥ 0.757 mg × 10^−6^. The outcome (median [IQR] 6 months mRS 2 [1,2,3,4,5] vs. 4 [1,2,3,4,5,6]; *p* = 0.003) in patients with CLR < 0.757 mg × 10^−6^ was better than that in patients with CLR ≥ 0.757 mg × 10^−6^.

## 4. Discussion

Accurate prognosis prediction for aSAH patients is necessary for determining the choices of clinical treatment strategies. The admission WBC, platelet, neutrophil, lymphocyte, and monocyte counts, and the levels of CRP are easy-to-quantify parameters and have promising predictive value for the outcome prediction after aSAH. In addition, CVS, DCI, and CH are the most critical post-aSAH complications, which are significantly associated with the outcome of aSAH. However, the predictive value of CLR for neurological outcome and complications was rarely investigated in patients with aSAH. In our study, we investigated admission CLR for the prediction of neurological functional outcome and complications after aSAH and found that high admission CLR was an independent risk factor for CVS and DCI after aSAH. The AUC of admission CLR for the predicted poor outcome (6 months mRS 3–6), CVS, DCI, and CH were 0.639, 0.834, 0.679, and 0.628, revealing that admission CLR had a favorable predictive value for CVS after aSAH. The sensitivity and specificity of admission CLR for CVS prediction were 77.1% and 75.4%. An admission CLR of 0.757 mg × 10^−6^ was identified as the best cutoff threshold to discriminate between CVS and non-CVS.

Elevated CLR was equal to high levels of CRP and relevant low serum levels of lymphocyte. CRP was identified in the 1930s and has been reported as an acute phase plasma protein and a response for the acute phase, which is synthesized in hepatocytes and induced by cytokines, particularly interleukin-6 (IL-6), which activates the complement system contributing to natural immunity and released into the blood [13,20,21]. CRP can rise 1000-fold within a few hours after the onset of stimuli such as infection, tissue necrosis, trauma, cancer, or various inflammatory diseases. The level of CRP rises to a peak 48 h after the initial stimulus and falls to baseline levels within 7–12 days when the inflammatory stimulus has disappeared [22,23]. CRP is also stimulated by interleukin-1 (IL-1), which is correlated with the pathogenesis of CVS [22,24,25]. According to the description of Fountas et al., elevated CRP levels in serum and cerebrospinal fluid (CSF) after angiographic vasospasm occurred in patients with aSAH, and higher CRP level measurements were strongly correlated with worse clinical outcomes in their cohort [22]. Additionally, Gaastra et al. confirmed that on-admission CRP levels are an independent predictive factor for the postoperative functional outcome after aSAH and high levels of on-admission CRP are closely correlated to poor functional outcomes after aSAH [20]. Romero et al. clarified that high serum CRP predicts unfavorable clinical outcomes and the occurrence of CVS and delayed ischemic neurological deficits (DIND) [26]. Meanwhile, when the central nervous system is stimulated, the immune system is activated, releasing large numbers of lymphocytes to reduce the damage to brain tissue through antigen recognition, cell activation, and immunocide [27]. Frösen et al. histologically compared 42 ruptured aneurysms with 24 unruptured aneurysms and found that lymphocytes were actively involved in the inflammatory cascade in the aneurysmal vessel wall [28]. When an aneurysm ruptures, lymphocytes are overconsumed, leading to a decrease in lymphocyte count, which is also considered to be associated with worsening brain injury and poor clinical outcomes [27]. In our study, the level of CLR in patients with poor outcome (6 months mRS 3–6) after aSAH was significantly higher than that in patients with good outcome (6 months mRS 0–2). CLR is a novel inflammatory biomarker and has been employed to serve as one of the most effective prognostic markers for pancreatic and rectal cancer surgery, tumor, and strangulated abdominal hernia (intestinal ischemia) [29,30,31,32]. Fan et al. found that CLR displayed higher predicted accuracy of poor prognosis compared to the combination of NLR, platelet to lymphocyte ratio (PLR), C-reactive protein to albumin ratio (CAR), neutrophil to albumin ratio (NAR), and platelet to albumin ratio (PAR) in patients with pancreatic cancer [30]. Recently, Zhang et al. first demonstrated that admission CLR serves as a valuable serum biomarker to predict the clinical prognosis of patients with aSAH [14]. In this study, increased admission levels of CLR were related to increased risk of poor outcome, which is consistent with the previously reported findings. Additionally, we first revealed that increased admission levels of CLR were associated with a high risk of occurrence of CVS, DCI, and CH. Higher AUC of CLR compared with CRP, WBC, neutrophil, and lymphocyte was observed to predict the occurrence of CVS in patients with aSAH. In addition, we first confirmed that admission CLR was independently associated with the occurrence of CVS and DCI after aSAH. Admission CLR had a favorable predictive value for CVS compared with poor outcome (6 months mRS 3–6) and DCI. High levels of CLR were associated with the poor prognosis of patients with aSAH and might be mediated by the elevated occurrence of CVS.

In addition, WBC represents the systemic inflammation status of humans and is widely investigated to predict the prognosis of diseases, such as cancers and aSAH [33,34]. Mahta et al. revealed that WBC count in the early course of SAH may have prognostic values in predicting DCI and functional outcome [35]. Furthermore, Geraghty et al. demonstrated that the WBC count within 24 h of admission was an independent risk factor for CVS after aSAH [35]. Similarly, Hu et al. reported that elevated WBC count within 24 h of admission could predict DCI occurrence between 4 and 30 days after aSAH [36]. In our study, the on-admission WBC count in patients with poor outcome (6 months mRS 3–6), CVS, and CH was significantly higher compared with patients with good outcome (6 months mRS 0–2), non-CVS and non-CH after aSAH. However, in our study, on-admission WBC count was not independently associated with poor outcome (6 months mRS 3–6) and the occurrence of CVS, DCI, and CH after aSAH. Furthermore, platelets are widely recognized as the major cells regulating hemostasis and thrombus formation. The influence of platelets on blood vessels has been attributed to their main role in thrombus formation, mediating myocardial infarction, stroke, and venous thromboembolism [37]. Wang et al. and Zhang et al. confirmed that platelets were correlated with the CVS and DCI after aSAH [13,38]. However, in our study, no significant differences in on-admission platelet count were observed in patients with poor outcome (6 months mRS 3–6), CVS, DCI, and CH compared to those with good outcome (6 months mRS 0–2), non-CVS, non-DCI, and non-CH after aSAH. The associations between on-admission WBC and platelet count and neurological functional outcome and complications after aSAH need to be further elucidated.

Additionally, an accurate prediction of clinical functional outcome and complications in patients with aSAH is still a challenge today. Admission CRP was widely employed to investigate the outcome and complications prediction, while admission CLR was rarely reported previously. We were the first to evaluate the predictive value of admission CLR for neurological functional outcome and complications after aSAH and found that the sensitivity of admission CLR in predicting CVS was higher than that compared with admission CRP (CLR [77.1%] vs. CRP [68.8%]). The sensitivity (CLR [77.1%] vs. CRP [68.8%]) and specificity (CLR [75.4%] vs. CRP [88.1%]) of CLR for CVS predicted were performed at relatively equal levels compared with admission CRP. We can combine the admission CLR and CRP to improve the sensitivity and specificity of CVS prediction. Moreover, in multivariate logistic regression analysis, admission CLR was an independent risk factor of CVS compared with admission NLR and MLR. Admission CLR displayed a better specificity for the prediction of CVS compared with admission NLR, PLR, and MLR. Our results supported the possibility of admission CLR being a promising predictive biomarker, which could provide some guidance for clinical work. Up to now, there is an abundance of lab parameters/ratios predicting outcome or DCI. We recruited C-reactive protein to lymphocyte ratio (CLR), neutrophil to lymphocyte ratio (NLR), platelet to lymphocyte ratio (PLR), and monocyte to lymphocyte ratio (MLR) in our research and found that these lab parameters/ratios improve the predictive value of CVS (CLR AUC = 0.834 vs. CRP AUC = 0.831; NLR AUC = 0.665 vs. neutrophil AUC = 0.633; PLR AUC = 0.624 vs. platelet AUC = 0.514; MLR AUC = 0.659 vs. monocyte AUC = 0.541; Figure 2; Table 4), DCI (CLR AUC = 0.679 vs. CRP AUC = 0.674; NLR AUC = 0.621 vs. neutrophil AUC = 0.599; PLR AUC = 0.545 vs. platelet AUC = 0.428; MLR AUC = 0.624 vs. monocyte AUC = 0.564; Supplemental Figure S2; Supplemental Table S8), CH (CLR AUC = 0.628 vs. CRP AUC = 0.593; NLR AUC = 0.681 vs. neutrophil AUC = 0.685; PLR AUC = 0.592 vs. platelet AUC = 0.425; MLR AUC = 0.622 vs. monocyte AUC = 0.553; Supplemental Figure S3; Supplemental Table S9), and poor outcome (6 months mRS 3–6) (CLR AUC = 0.639 vs. CRP AUC = 0.610; NLR AUC = 0.644 vs. neutrophil AUC = 0.610; PLR AUC = 0.582 vs. platelet AUC = 0.461; MLR AUC = 0.654 vs. monocyte AUC = 0.565; Supplemental Figure S1; Supplemental Table S7). In the future, more and more novel and accurate predictive biomarkers will be developed to predict the prognosis and complications in patients with aSAH.

Our study was explorative in nature. The explorative approach was deliberately chosen due to the complex and multifactorial nature of CVS in aSAH. This condition is influenced by a range of biological and physiological factors, making it challenging to hypothesize the impact of specific biomarkers a priori. We aimed to comprehensively investigate the potential of CLR as a predictive biomarker, considering its previously demonstrated relevance in inflammatory and vascular conditions. Selection of parameters: The parameters analyzed in this study, including some less common ones, were carefully chosen based on their potential relevance to the pathophysiology of CVS. Each parameter was selected based on a thorough review of existing literature and our clinical experience in managing aSAH patients. We believe that examining these parameters, in conjunction with CLR, provided a more robust understanding of the biomarker’s predictive capabilities. Hypothesis development: While our approach was explorative, it was not without direction. The hypothesis that CLR could serve as a predictive biomarker for CVS in aSAH was based on preliminary data and existing research indicating the role of inflammation and immune response in the pathogenesis of CVS. The additional parameters were included to investigate the specificity and sensitivity of CLR in this context and to explore potential mechanisms that might underlie its predictive value. Statistical analysis: To ensure the rigor of our explorative approach, we employed robust statistical methods. These included the use of multivariate analyses to control for potential confounders and to isolate the specific impact of CLR on the risk of CVS. In conclusion, the explorative nature of our study was a deliberate and considered choice, aimed at thoroughly investigating CLR’s potential as a predictive biomarker in aSAH. The parameters analyzed were selected based on their relevance to the disease’s pathophysiology and the hypothesis was grounded in existing scientific evidence. We believe our findings contribute valuable insights into the role of CLR in aSAH and pave the way for further hypothesis-driven research in this area.

Due to the complex nature of the disease, every non-invasive predictive marker may add its diagnostic value providing in this case an easy-to-quantify ratio to improve the predictive value of current diagnostics for post hemorrhagic complications. Risk stratification: The primary value of CLR in this context is its ability to stratify patients based on their risk of developing CVS. Clinicians can prioritize monitoring and interventions for the patients who are most likely to benefit from them. Tailoring clinical monitoring: For patients with a high CLR, indicating a higher risk of CVS, clinicians can intensify monitoring protocols including more frequent neurological assessments, more frequent monitoring of hemodynamic parameters, and earlier or more frequent imaging tests to detect vasospasm before causing clinical deterioration. Early intervention and preventive strategies: Stratifying a patient’s risk can guide the initiation of preventive measures. For instance, clinicians might be more vigilant about implementing strategies like hemodynamic augmentation or prophylactic use of nimodipine in patients with elevated CLR. Customizing treatment plans: The CLR value can help in deciding the aggressiveness of therapeutic interventions. Clinicians might choose earlier endovascular treatments for vasospasm (like angioplasty or intra-arterial nimodipine therapy) rather than waiting for clinical deterioration in patients with high CLR. Patient counseling and resource allocation: Understanding the risk of CVS can assist in patient and family counseling regarding prognosis and potential complications. It also helps in the optimal allocation of healthcare resources, such as ICU and specialized care for those who need it most. Research and clinical trials: The identification of CLR as a predictive biomarker opens directions for further research, including clinical trials to test targeted interventions for patients identified as high-risk based on the levels of CLR. In conclusion, the addition of CLR as a predictive biomarker for CVS in aSAH patients is not just about adding another test; it is about providing a practical, cost-effective tool for better patient stratification and management. It helps to guide clinical decisions from monitoring to intervention and has the potential to significantly improve patient outcomes.

## 5. Limitations

Admittedly, some limitations exist in our research. First, our study was a retrospective and single-center study making it susceptible to bias arising from patient and treatment choices. Therefore, prospective multi-center clinical trials on the associations of CLR with neurological functional outcome and complications after aSAH need to be set up. Second, the aSAH patients diagnosed with the diseases influenced by the peripheral serum biomarkers were excluded. It is still not clear whether the CLR has a predictive value for the prognosis and the occurrence of neurological complications in patients with admission systemic metabolic diseases. The development of new predictive biomarkers and models for patients with admission systemic metabolic diseases is essential. Third, due to 284 cases not having complete routine blood tests, our results strongly recommend that clinicians should complete routine blood and comprehensive biochemical tests within 24 h at the hospital emergency unit for every aSAH patient. Fourth, out of 662 patients, only 166 patients included making it susceptible to sampling bias. Last but not least, though we reported the associations of on-admission serum biomarkers with neurological functional outcome and complications after aSAH, the potential mechanisms are still not fully understood.

## 6. Conclusions

In summary, admission serum levels of CLR in patients with CVS, poor outcome (6 months mRS 3–6), DCI, and CH were significantly higher than those in patients with good outcome (6 months mRS 0–2), non-CVS, non-DCI, and non-CH. The on-admission high levels of CLR served as an independent risk factor for CVS and DCI. Admission CLR is an easy-to-quantify laboratory parameter that efficiently predicts the CVS after aSAH, which could provide some guidance for clinicians to evaluate for possible progression and treatment strategies in patients with aSAH.

## Figures and Tables

**Table 1:** Patient characteristics.

Variables	Patients (*n* = 166)
Demographics	
Age, mean (SD), y	55.58 ± 12.22
Female sex, *n* (%)	114 (68.7)
Medical history, *n* (%)	
Hypertension	117 (70.5)
Admission status, median (IQR)	
WFNS grade	2 (1–5)
GCS score	13 (4–15)
Admission serum biomarkers, median (IQR)	
CRP, (mg/dL)	0.50 (0.20–1.40)
WBC, (×10^9^/L)	13.35 (10.58–15.90)
Platelet, (×10^9^/L)	230.50 (196.75–278.25)
Neutrophil, (×10^9^/L)	11.17 (8.52–14.00)
Lymphocyte, (×10^9^/L)	1.05 (0.74–1.60)
Monocyte, (×10^9^/L)	0.78 (0.50–1.00)
CLR, (mg × 10^−6^)	0.50 (0.17–1.56)
NLR	11.00 (6.24–16.74)
PLR	218.87 (143.57–323.11)
MLR	0.63 (0.43–0.90)
Neuroradiological data	
mFisher score, median (IQR)	4 (3–4)
Intracerebral hemorrhage, *n* (%)	37 (22.3)
Subdural hemorrhage, *n* (%)	14 (8.4)
Aneurysmal locations, *n* (%)	
ACA/ACOM	77 (46.4)
MCA	38 (22.9)
PCOM	19 (11.4)
ICA	9 (5.4)
PC	23 (13.9)
Aneurysmal sizes, *n* (%)	
0–4.9 mm	49 (29.5)
5–6.9 mm	46 (27.7)
7–9.9 mm	21 (12.7)
10–19.9 mm	19 (11.4)
≥20 mm	6 (3.6)
Missing	25 (15.1)
Treatment status, *n* (%)	
Coil	46 (27.7)
Clip	114 (68.7)
No treatment	6 (3.6)
Neurological complications, *n* (%)	
CVS	48 (28.9)
DCI	53 (31.9)
CH	88 (53.0)
Seizures	23 (13.9)
Neurological functional outcome	
6 months mRS, median (IQR)	3 (1–5)

SD: standard deviation, IQR: interquartile range, WFNS: World Federation of Neurosurgical Societies, GCS Glasgow Coma Score, CRP: C-reactive protein, mg/dL: milligram/deciliter, mg/L: milligram/liter, WBC: white blood cell, ×10^9^/L ×10^9^/Liter, CLR: C-reactive protein to lymphocyte ratio, NLR: neutrophil to lymphocyte ratio, PLR: platelet to lymphocyte ratio, MLR: monocyte to lymphocyte ratio, mFisher: modified Fisher, ACA: anterior cerebral artery, ACOM: anterior communicating artery, MCA: middle cerebral artery, PCOM: posterior communicating artery, ICA: internal carotid artery, PC: posterior circulation, CVS: cerebral vasospasm, DCI: delayed cerebral ischemia, CH: chronic hydrocephalus, mRS: modified Rankin Scale.

**Table 2:** Univariate analysis on CVS after aSAH.

Variables	CVS (*n* = 48)	Non-CVS (*n* = 118)	*p* Value
Demographics			
Age, mean (SD), y	57.60 ± 10.40	54.75 ± 12.83	0.174
Female sex, *n* (%)	33 (68.8)	81 (68.6)	0.989
Medical history, *n* (%)			
Hypertension	37 (77.1)	80 (67.8)	0.234
Admission status, median (IQR)			
WFNS grade	3 (2–5)	2 (1–5)	0.018 *
GCS score	11 (3–14)	14 (5–15)	0.016 *
Admission serum biomarkers, median (IQR)			
CRP, (mg/dL)	1.95 (0.80–3.10)	0.30 (0.18–0.80)	<0.001 *
WBC, (×10^9^/L)	14.05 (11.68–18.33)	12.60 (10.10–15.70)	0.040 *
Platelet, (×10^9^/L)	237.00 (194.25–284.50)	226.00 (199.25–274.25)	0.781
Neutrophil, (×10^9^/L)	11.95 (10.05–15.95)	10.49 (7.88–13.49)	0.007 *
Lymphocyte, (×10^9^/L)	0.93 (0.60–1.32)	1.17 (0.80–1.62)	0.010 *
Monocyte, (×10^9^/L)	0.80 (0.52–1.18)	0.73 (0.50–1.00)	0.404
CLR, (mg × 10^−6^)	1.83 (0.77–4.46)	0.30 (0.12–0.76)	<0.001 *
NLR	12.90 (9.80–20.59)	8.74 (5.54–15.97)	0.001 *
PLR	262.08 (189.38–397.88)	215.88 (135.60–294.97)	0.012 *
MLR	0.74 (0.59–1.11)	0.58 (0.39–0.77)	0.001 *
Neuroradiological data			
mFisher score, median (IQR)	4 (3–4) 3.56 ± 0.65	4 (3–4) 3.43 ± 0.63	0.151
Intracerebral hemorrhage, *n* (%)	12 (25.0)	25 (21.2)	0.592
Subdural hemorrhage, *n* (%)	3 (6.3)	11 (9.3)	0.736
Aneurysmal locations, *n* (%)			0.248
ACA/ACOM	19 (39.6)	58 (49.2)	
MCA	9 (18.8)	29 (24.6)	
PCOM	9 (18.8)	10 (8.5)	
ICA	2 (4.2)	7 (5.9)	
PC	9 (18.8)	14 (11.9)	
Aneurysmal sizes, *n* (%)			0.282
0–4.9 mm	13 (27.1)	36 (30.5)	
5–6.9 mm	11 (22.9)	35 (29.7)	
7–9.9 mm	4 (8.3)	17 (14.4)	
10–19.9 mm	9 (18.8)	10 (8.5)	
≥20 mm	3 (6.3)	3 (2.5)	
Missing	8 (16.7)	17 (14.4)	
Treatment status, *n* (%)			0.903
Coil	14 (29.2)	32 (27.1)	
Clip	32 (66.7)	82 (69.5)	
No treatment	2 (4.2)	4 (3.4)	
Neurological complications, *n* (%)			
DCI	27 (56.3)	26 (22.0)	<0.001 *
CH	35 (72.9)	53 (44.9)	0.001 *
Seizures	11 (22.9)	12 (10.2)	0.031 *
Neurological functional outcome			
6 months mRS, median (IQR)	5 (2–6)	2 (1–5)	<0.001 *

CVS: cerebral vasospasm, aSAH: aneurysmal subarachnoid hemorrhage, SD: standard deviation, IQR: interquartile range, WFNS: World Federation of Neurosurgical Societies, GCS: Glasgow Coma Score, CRP C-reactive protein, mg/dL: milligram/deciliter, mg/L: milligram/liter, WBC: white blood cell, ×10^9^/L ×10^9^/Liter, CLR: C-reactive protein to lymphocyte ratio, NLR: neutrophil to lymphocyte ratio, PLR: platelet to lymphocyte ratio, MLR: monocyte to lymphocyte ratio, mFisher: modified Fisher, ACA: anterior cerebral artery, ACOM: anterior communicating artery, MCA: middle cerebral artery, PCOM: posterior communicating artery, ICA: internal carotid artery, PC: posterior circulation, DCI: delayed cerebral ischemia, CH: chronic hydrocephalus, mRS: modified Rankin Scale, * *p* < 0.05 are considered significant.

**Table 3:** Multivariate logistic regression analysis of variables associated with CVS after aSAH.

Variables		CVS (*n* = 48)	
	OR	95% CI	*p* Value
CLR	2.116	1.507–2.971	<0.001 *
NLR	1.007	0.946–1.072	0.830
MLR	0.843	0.372–1.909	0.682

CVS: cerebral vasospasm, aSAH: aneurysmal subarachnoid hemorrhage, OR: odds ratio, CI: confidence interval, CLR: C-reactive protein to lymphocyte ratio, NLR: neutrophil to lymphocyte ratio, MLR: monocyte to lymphocyte ratio, * *p* < 0.05 are considered significant.

**Table 4:** Comparison of the predictive value of different admission serum biomarkers associated with CVS.

Predictive Biomarkers	AUC	95% CI	Sensitivity (%)	Specificity (%)	Cut-Off Value	*p* Value
CLR	0.834	0.767–0.901	77.1	75.4	0.757 mg × 10^−6^	<0.001 *
NLR	0.665	0.577–0.752	85.4	50.8	8.775	0.001 *
PLR	0.624	0.531–0.718	81.3	43.2	177.312	0.012 *
MLR	0.659	0.571–0.748	77.1	51.7	0.584	0.001 *
CRP	0.831	0.758–0.903	68.8	88.1	1.150 mg/dL	<0.001 *
WBC	0.602	0.508–0.697	72.9	47.5	12.050 × 10^9^/L	0.040 *
Platelet	0.514	0.416–0.612	31.3	78.8	280.500 × 10^9^/L	0.781
Neutrophil	0.633	0.542–0.723	72.9	53.4	10.840 × 10^9^/L	0.007 *
Lymphocyte	0.373	0.280–0.465	100	0	–	0.010 *
Monocyte	0.541	0.442–0.641	37.5	74.6	0.980 × 10^9^/L	0.405

CVS: cerebral vasospasm, AUC: area under the curve, CI: confidence interval, CLR: C-reactive protein to lymphocyte ratio, NLR: neutrophil to lymphocyte ratio, PLR: platelet to lymphocyte ratio, MLR: monocyte to lymphocyte ratio, CRP: C-reactive protein, WBC: white blood cell, * *p* < 0.05 are considered significant.

**Table 5:** Neurological complications and functional outcome categorized by the identified CLR threshold (0.757 mg × 10^−6^).

Variables	CLR		*p* Value
	<0.757 mg × 10^−6^ (*n* = 100)	≥0.757 mg × 10^−6^ (*n* = 66)	
Neurological complications, *n* (%)			
CVS	11 (11.0)	37 (56.1)	<0.001 *
DCI	20 (20.0)	33 (50.0)	<0.001 *
CH	45 (45.0)	43 (65.2)	0.011 *
Seizures	14 (14.0)	9 (13.6)	0.947
Neurological functional outcome			
6 months mRS, median (IQR)	2 (1–5)	4 (1–6)	0.003 *

CLR: C-reactive protein to lymphocyte ratio, CVS: cerebral vasospasm, DCI: delayed cerebral ischemia, CH: chronic hydrocephalus, IQR: interquartile range, mRS: modified Rankin Scale, * *p* < 0.05 are considered significant.

## Data Availability

Available upon reasonable request.

## Supplementary Materials

The following supporting information can be downloaded at: https://www.mdpi.com/article/10.3390/jcm13040940/s1, STROBE checklist; Table S1. Univariate analysis on outcome (6 months mRS) after aSAH. Table S2. Univariate analysis on DCI after aSAH. Table S3. Univariate analysis on CH after aSAH. Table S4. Multivariate logistic regression analysis of variables associated with poor outcome (6 months mRS 3–6). Table S5. Multivariate logistic regression analysis of variables associated with DCI after aSAH. Table S6. Multivariate logistic regression analysis of variables associated with CH after aSAH. Table S7. Comparison of predictive value of different admission serum biomarkers associated with poor outcome (6 months mRS 3–6). Table S8 Comparison of predictive value of different admission serum biomarkers associated with DCI. Table S9 Comparison of predictive value of different admission serum biomarkers associated with CH. Figure S1. Predictive value of each admission serum biomarkers for poor outcome (6 months mRS 3–6) using receiver operating characteristics (ROC) curve. Figure S2. Predictive value of each admission serum biomarkers for delayed cerebral ischemia (DCI) using receiver operating characteristics (ROC) curve. Figure S3. Predictive value of each admission serum biomarkers for chronic hydrocephalus (CH) using receiver operating characteristics (ROC) curve.

## Abbreviations

SAH: subarachnoid hemorrhage, aSAH: aneurysmal subarachnoid hemorrhage, CVS: cerebral vasospasm, DCI: delayed cerebral ischemia, CH: chronic hydrocephalus, TCD: transcranial Doppler ultrasonography, DSA: digital subtraction angiography, CTA: computed tomography angiography, CTP: computed tomography perfusion, WBC: white blood cell, NLR neutrophil to lymphocyte ratio, CRP: C-reactive protein, CLR: C-reactive protein to lymphocyte ratio, CT: computed tomography, STROBE: Strengthening the Reporting of Observational Studies in Epidemiology, mFisher: modified Fisher, WFNS: World Federation of Neurosurgical Societies, GCS: Glasgow Coma Score, NICU: Neurological Intensive Care Unit, MRI magnetic resonance imaging, mRS: modified Rankin Scale, PLR: platelet to lymphocyte ratio, MLR: monocyte to lymphocyte ratio, SD standard deviation, IQR: interquartile range, OR: odds ratio, CI: confidence interval, ROC: receiver operating characteristic, ACA anterior cerebral artery, ACOM: anterior communicating artery, MCA: middle cerebral artery, PCOM: posterior communicating artery, ICA: internal carotid artery, PC: posterior circulation, mg/d:L milligram/deciliter, mg/L milligram/liter, ×10^9^/L ×10^9^/Liter, AUC: area under the curve, IL-6: interleukin-6, IL-1: interleukin-1, CSF: cerebrospinal fluid, DIND: delayed ischemic neurological deficits; PLR: platelet to lymphocyte ratio, CAR C-reactive protein to albumin ratio, NAR: neutrophil to albumin ratio, PAR: platelet to albumin ratio.
