# Supplementary material for: CLR (C-Reactive Protein to Lymphocyte Ratio) Served as a Promising Predictive Biomarker for Cerebral Vasospasm in Aneurysmal Subarachnoid Hemorrhage (aSAH): A Retrospective Cohort Study
Source: J Clin Med. 2024 Feb 6;13(4):940. doi: 10.3390/jcm13040940 (PMC10889261; doi:10.3390/jcm13040940)
Supplement: Supplementary file 1 [file jcm-13-00940-s001.zip › jcm-2814020-supplementary.pdf]

CLR (C-Reactive Protein to Lymphocyte Ratio) Served as a Promising Predictive Biomarker for Cerebral Vasospasm in Aneurysmal Subarachnoid Hemorrhage (aSAH): A Retrospective Cohort Study

Ke Li [1], Dilaware Khan [1], Igor Fischer [1], Daniel Hänggi [2,3], Jan F. Cornelius [1] and Sajjad Muhammad [1,2,4,*]

[1] Department of Neurosurgery, Medical Faculty and University Hospital Düsseldorf, Heinrich-Heine-University Düsseldorf, 40225 Düsseldorf, Germany; ke.li@uni-duesseldorf.de (K.L.); dilaware.khan@med.uni-duesseldorf.de (D.K.); igor.fischer@med.uni-duesseldorf.de (I.F.); cornelius@med.uni-duesseldorf.de (J.F.C.)
[2] Department of Neurosurgery, King Edward Medical University, Lahore 54000, Pakistan; haenggi-assistant@ini-hannover.de
[3] Department of Neurosurgery, International Neuroscience Institute, 30625 Hannover, Germany
[4] Department of Neurosurgery, University of Helsinki, Helsinki University Hospital, 00290 Helsinki, Finland
* Correspondence: sajjad.muhammad@med.uni-duesseldorf.de; Tel.: +492118107823

Supplemental Files

Table of contents

Supplemental Table S1 - Page 2
Supplemental Table S2 - Page 4
Supplemental Table S3 - Page 6
Supplemental Table S4 - Page 8
Supplemental Table S5 - Page 9
Supplemental Table S6 - Page 10
Supplemental Figure S1 Page 11
Supplemental Table S7 - Page 12
Supplemental Figure S2 Page 13
Supplemental Table S8 - Page 14
Supplemental Figure S3 Page 15
Supplemental Table S9 - Page 16

Supplemental Table S1 Univariate analysis on outcome (6 months mRS) after aSAH

Variables	mRS (0–2) (n = 82)	mRS (3–6) (n = 84)	*p* value
Demographics			
Age, mean (SD), y	51.74±11.54	59.32±11.74	<0.001*
Female sex, n (%)	49 (59.8)	65 (77.4)	0.014*
Medical history, n (%)			
Hypertension	55 (67.1)	62 (73.8)	0.341
Admission status, median (IQR)			
WFNS grade	1 (1–2)	5 (2–5)	<0.001*
GCS score	15 (14–15)	4 (3–13)	<0.001*
Admission serum biomarkers, median (IQR)			
CRP, (mg/dL)	0.40 (0.20–0.90)	0.75 (0.20–2.43)	0.014*
WBC, ($\times10^9$/L)	12.20 (10.00–14.70)	14.20 (11.25–17.30)	0.012*
Platelet, ($\times10^9$/L)	237.50 (208.50–279.75)	227.00 (184.50–277.25)	0.381
Neutrophil, ($\times10^9$/L)	10.44 (7.90–12.73)	12.05 (9.12–15.09)	0.014*
Lymphocyte, ($\times10^9$/L)	1.15 (0.85–1.67)	0.95 (0.60–1.56)	0.018*
Monocyte, ($\times10^9$/L)	0.72 (0.49–0.94)	0.80 (0.56–1.09)	0.146
CLR, (mg$\times10^{-6}$)	0.34 (0.13–0.83)	0.77 (0.20–2.74)	0.002*
NLR	8.74 (5.32–13.32)	12.75 (7.11–19.17)	0.001*
PLR	206.73 (140.36–294.97)	238.72 (156.84–362.25)	0.069
MLR	0.55 (0.39–0.74)	0.70 (0.51–1.12)	0.001*
Neuroradiological data			
mFisher score, median (IQR)	3 (3–4)	4 (3–4)	<0.001*
Intracerebral hemorrhage, n (%)	7 (8.5)	30 (35.7)	<0.001*
Subdural hemorrhage, n (%)	3 (3.7)	11 (11.3)	0.029*
Aneurysmal locations, n (%)			0.355
ACA/ACOM	42 (51.2)	35 (41.7)	
MCA	19 (23.2)	19 (22.6)	
PCOM	10 (12.2)	9 (10.7)	
ICA	4 (4.9)	5 (6.0)	
PC	7 (8.5)	16 (19.0)	
Aneurysmal sizes, n (%)			0.006*
0–4.9 mm	27 (32.9)	22 (26.2)	
5–6.9 mm	27 (32.9)	19 (22.6)	
7–9.9 mm	14 (17.1)	7 (8.3)	
10–19.9 mm	6 (7.3)	13 (15.5)	

≥20 mm	0	6 (7.1)	
Missing	8 (9.8)	17 (20.0)	
Treatment status, n (%)			0.016*
Coil	20 (24.4)	26 (31.0)	
Clip	62 (75.6)	52 (61.9)	
No treatment	0	6 (7.1)	
Neurological complications, n (%)			
CVS	13 (15.9)	35 (41.7)	<0.001*
DCI	9 (11.0)	44 (52.4)	<0.001*
CH	26 (31.7)	62 (73.8)	<0.001*
Seizures	9 (11.0)	14 (16.7)	0.289

mRS modified Rankin Scale; *aSAH* aneurysmal subarachnoid hemorrhage, *SD* standard deviation, *IQR* interquartile range, *WFNS* World Federation of Neurosurgical Societies, *GCS* Glasgow Coma Score, *CRP* C-reactive protein, *mg/dL* milligram/deciliter, *mg/L* milligram/liter, *WBC* white blood cell, $\times 10^9/L$ $\times 10^9$/Liter, *CLR* C-reactive protein to lymphocyte ratio, *NLR* neutrophil to lymphocyte ratio, *PLR* platelet to lymphocyte ratio, *MLR* monocyte to lymphocyte ratio, *mFisher* modified Fisher, *ACA* anterior cerebral artery, *ACOM* anterior communicating artery, *MCA* middle cerebral artery, *PCOM* posterior communicating artery, *ICA* internal carotid artery, *PC* posterior circulation, *CVS* cerebral vasospasm, *DCI* delayed cerebral ischemia, *CH* chronic hydrocephalus, $^*p < 0.05$ are considered significant

Supplemental Table S2 Univariate analysis on DCI after aSAH

Variables	DCI (n = 53)	Non-DCI (n = 113)	*p* value
Demographics			
Age, mean (SD), y	59.68±11.98	53.65±11.89	0.003*
Female sex, n (%)	38 (71.7)	76 (67.3)	0.565
Medical history, n (%)			
Hypertension	40 (75.5)	77 (68.1)	0.334
Admission status, median (IQR)			
WFNS grade	4 (2–5)	2 (1–4)	<0.001*
GCS score	6 (3–13)	14 (8–15)	<0.001*
Admission serum biomarkers, median (IQR)			
CRP, (mg/dL)	1.60 (0.20–1.90)	0.40 (0.20–0.90)	<0.001*
WBC, ($\times10^9$/L)	14.20 (11.40–16.50)	12.60 (10.10–15.60)	0.056
Platelet, ($\times10^9$/L)	226.00 (183.00–270.00)	233.00 (206.00–285.50)	0.138
Neutrophil, ($\times10^9$/L)	12.10 (9.16–14.62)	10.83 (7.95–13.73)	0.041*
Lymphocyte, ($\times10^9$/L)	0.97 (0.61–1.50)	1.11 (0.80–1.60)	0.083
Monocyte, ($\times10^9$/L)	0.80 (0.62–1.09)	0.72 (0.50–1.00)	0.181
CLR, (mg$\times10^{-6}$)	1.34 (0.21–3.60)	0.43 (0.13–0.84)	<0.001*
NLR	12.76 (8.14–22.39)	9.33 (5.90–15.85)	0.012*
PLR	223.48 (150.13–415.17)	216.50 (142.31–316.86)	0.350
MLR	0.69 (0.56–1.32)	0.58 (0.42–0.81)	0.010*
Neuroradiological data			
mFisher score, median (IQR)	4 (3–4)	3 (3–4)	0.031*
Intracerebral hemorrhage, n (%)	19 (35.8)	18 (15.9)	0.004*
Subdural hemorrhage, n (%)	8 (15.1)	6 (5.3)	0.034*
Aneurysmal locations, n (%)			0.079
ACA/ACOM	17 (32.1)	60 (53.1)	
MCA	17 (32.1)	21 (18.6)	
PCOM	8 (15.1)	11 (9.7)	
ICA	2 (3.8)	7 (6.2)	
PC	9 (17.0)	14 (12.4)	
Aneurysmal sizes, n (%)			0.012*
0–4.9 mm	13 (24.5)	36 (31.9)	
5–6.9 mm	9 (17.0)	37 (32.7)	
7–9.9 mm	5 (9.4)	16 (14.2)	
10–19.9 mm	9 (17.0)	10 (8.8)	

≥20 mm	4 (7.5)	2 (1.8)	
Missing	13 (24.5)	12 (10.6)	
Treatment status, n (%)			0.152
Coil	10 (18.9)	36 (31.9)	
Clip	40 (75.5)	74 (65.5)	
No treatment	3 (5.7)	3 (2.7)	
Neurological complications, n (%)			
CVS	27 (50.9)	21 (18.6)	<0.001*
CH	39 (73.6)	49 (43.9)	<0.001*
Seizures	8 (15.1)	15 (13.3)	0.752
Neurological functional outcome			
6 months mRS, median (IQR)	5 (4–6)	2 (1–3)	<0.001*

DCI delayed cerebral ischemia, *aSAH* aneurysmal subarachnoid hemorrhage, *SD* standard deviation, *IQR* interquartile range, *WFNS* World Federation of Neurosurgical Societies, *GCS* Glasgow Coma Score, *CRP* C-reactive protein, *mg/dL* milligram/deciliter, *mg/L* milligram/liter, *WBC* white blood cell, $\times 10^9/L$ $\times 10^9$/Liter, *CLR* C-reactive protein to lymphocyte ratio, *NLR* neutrophil to lymphocyte ratio, *PLR* platelet to lymphocyte ratio, *MLR* monocyte to lymphocyte ratio, *mFisher* modified Fisher, *ACA* anterior cerebral artery, *ACOM* anterior communicating artery, *MCA* middle cerebral artery, *PCOM* posterior communicating artery, *ICA* internal carotid artery, *PC* posterior circulation, *CVS* cerebral vasospasm, *CH* chronic hydrocephalus, *mRS* modified Rankin Scale, $^*p < 0.05$ are considered significant

Supplemental Table S3 Univariate analysis on CH after aSAH

Variables	CH (n = 88)	Non-CH (n = 78)	*p* value
Demographics			
Age, mean (SD), y	56.26±12.96	54.81±11.35	0.446
Female sex, n (%)	66 (75.0)	48 (61.5)	0.062
Medical history, n (%)			
Hypertension	64 (72.7)	53 (67.9)	0.501
Admission status, median (IQR)			
WFNS grade	4 (2–5)	1 (1–3)	<0.001*
GCS score	9 (3–14)	15 (12–15)	<0.001*
Admission serum biomarkers, median (IQR)			
CRP, (mg/dL)	0.70 (0.20–1.90)	0.40 (0.20–0.90)	0.038*
WBC, ($\times 10^9$/L)	14.45 (11.93–16.85)	11.55 (9.58–13.83)	<0.001*
Platelet, ($\times 10^9$/L)	219.00 (187.75–273.75)	251.50 (209.75–282.25)	0.096
Neutrophil, ($\times 10^9$/L)	12.34 (10.26–14.74)	9.34 (7.41–11.71)	<0.001*
Lymphocyte, ($\times 10^9$/L)	0.95 (0.62–1.52)	1.20 (0.82–1.64)	0.010*
Monocyte, ($\times 10^9$/L)	0.80 (0.51–1.12)	0.73 (0.50–0.89)	0.241
CLR, (mg$\times 10^{-6}$)	0.75 (0.21–1.98)	0.33 (0.13–0.95)	0.004*
NLR	12.90 (8.63–19.17)	8.46 (4.83–13.05)	<0.001*
PLR	240.17 (163.58–352.87)	198.43 (132.50–296.56)	0.041*
MLR	0.69 (0.47–1.08)	0.56 (0.39–0.74)	0.007*
Neuroradiological data			
mFisher score, median (IQR)	4 (3–4)	3 (3–4)	<0.001*
Intracerebral hemorrhage, n (%)	29 (33.0)	8 (10.3)	<0.001*
Subdural hemorrhage, n (%)	9 (10.2)	5 (6.4)	0.377
Aneurysmal locations, n (%)			0.301
ACA/ACOM	36 (40.9)	41 (52.6)	
MCA	24 (27.3)	14 (17.9)	
PCOM	11 (12.5)	8 (10.3)	
ICA	3 (3.4)	6 (7.7)	
PC	14 (15.9)	9 (11.5)	
Aneurysmal sizes, n (%)			0.437
0–4.9 mm	27 (30.7)	22 (28.2)	
5–6.9 mm	21 (23.9)	25 (32.1)	
7–9.9 mm	9 (10.2)	12 (15.4)	
10–19.9 mm	12 (13.6)	7 (9.0)	

≥20 mm	5 (5.7)	1 (1.3)	
Missing	14 (15.9)	11 (14.1)	
Treatment status, n (%)			0.838
Coil	24 (27.2)	22 (28.2)	
Clip	60 (68.2)	54 (69.2)	
No treatment	4 (4.5)	2 (2.6)	
Neurological complications, n (%)			
CVS	35 (39.8)	13 (16.7)	0.001*
DCI	39 (44.3)	14 (17.9)	<0.001*
Seizures	14 (15.9)	9 (11.5)	0.416
Neurological functional outcome			
6 months mRS, median (IQR)	5 (2–6)	1 (1–3)	<0.001*

CH chronic hydrocephalus, *aSAH* aneurysmal subarachnoid hemorrhage, *SD* standard deviation, *IQR* interquartile range, *WFNS* World Federation of Neurosurgical Societies, *GCS* Glasgow Coma Score, *CRP* C-reactive protein, *mg/dL* milligram/deciliter, *mg/L* milligram/liter, *WBC* white blood cell, $\times 10^9/L$ $\times 10^9$/Liter, *CLR* C-reactive protein to lymphocyte ratio, *NLR* neutrophil to lymphocyte ratio, *PLR* platelet to lymphocyte ratio, *MLR* monocyte to lymphocyte ratio, *mFisher* modified Fisher, *ACA* anterior cerebral artery, *ACOM* anterior communicating artery, *MCA* middle cerebral artery, *PCOM* posterior communicating artery, *ICA* internal carotid artery, *PC* posterior circulation, *CVS* cerebral vasospasm, *DCI* delayed cerebral ischemia, *mRS* modified Rankin Scale, *$p < 0.05$ are considered significant

Supplemental Table S4 Multivariate logistic regression analysis of variables associated with poor outcome (6 months mRS 3-6)

Variables	mRS (3-6) (n = 84)		
	OR	95% CI	*p* value
Age	1.029	0.987–1.072	0.182
WFNS grade	0.705	0.254–1.958	0.502
GCS	0.676	0.481–0.949	0.024*
CLR	1.474	0.997–2.177	0.052
NLR	0.952	0.879–1.032	0.231
MLR	1.215	0.417–3.544	0.721
mFisher grade	1.547	0.648–3.696	0.326
Intracerebral hematoma	1.209	0.305–4.783	0.787
CVS	1.487	0.487–4.534	0.486
DCI	3.388	1.101–10.428	0.033*
CH	4.120	1.525–11.131	0.005*

mRS modified Rankin Scale, *OR* odds ratio, *CI* confidence interval, *WFNS* World Federation of Neurosurgical Societies, *GCS* Glasgow Coma Score, *CLR* C-reactive protein to lymphocyte ratio, *NLR* neutrophil to lymphocyte ratio, *MLR* monocyte to lymphocyte ratio, *mFisher* modified Fisher, *CVS* cerebral vasospasm, *DCI* delayed cerebral ischemia, *CH* chronic hydrocephalus, *$p < 0.05$ are considered significant

Supplemental Table S5 Multivariate logistic regression analysis of variables associated with DCI after aSAH

Variables	DCI (n = 53)		
	OR	95% CI	*p* value
Age	1.025	0.990–1.061	0.165
WFNS grade	1.345	0.588–3.078	0.483
GCS	0.968	0.747–1.254	0.805
CLR	1.594	1.220–2.084	0.001*
Intracerebral hematoma	1.090	0.433–2.742	0.854

DCI delayed cerebral ischemia,, *aSAH* aneurysmal subarachnoid hemorrhage, *OR* odds ratio, *CI* confidence interval, *WFNS* World Federation of Neurosurgical Societies, *GCS* Glasgow Coma Score, *CLR* C-reactive protein to lymphocyte ratio, *$p < 0.05$ are considered significant

Supplemental Table S6 Multivariate logistic regression analysis of variables associated with CH after aSAH

Variables	CH (n = 88)		
	OR	95% CI	*p* value
WFNS grade	1.749	0.785–3.896	0.171
GCS	1.113	0.862–1.437	0.411
WBC	1.348	0.872–2.083	0.180
Neutrophil	0.755	0.458–1.247	0.273
CLR	0.957	0.843–1.087	0.501
NLR	1.069	0.990–1.156	0.090
mFisher grade	1.893	0.956–3.749	0.067
Intracerebral hematoma	1.574	0.564–4.396	0.386

CH chronic hydrocephalus, *aSAH* aneurysmal subarachnoid hemorrhage, *OR* odds ratio, *CI* confidence interval, *WFNS* World Federation of Neurosurgical Societies, *GCS* Glasgow Coma Score, *WBC* white blood cell, *CLR* C-reactive protein to lymphocyte ratio, *NLR* neutrophil to lymphocyte ratio, *mFisher* modified Fisher, *$p < 0.05$ are considered significant

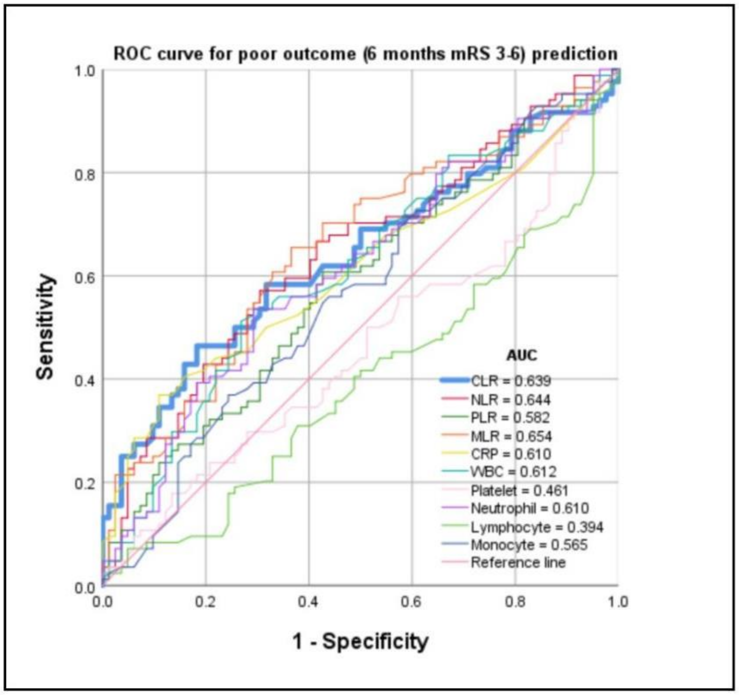

Supplemental Figure S1 Predictive value of each admission serum biomarkers for poor outcome (6 months mRS 3-6) using receiver operating characteristics (ROC) curve.

Supplemental Table S7 Comparison of predictive value of different admission serum biomarkers associated with poor outcome (6 months mRS 3-6).

Predictive biomarkers	AUC	95% CI	Sensitivity (%)	Specificity (%)	Cut-off value	*p* value
CLR	0.639	0.555–0.724	46.4	81.7	1.012 mg$\times10^{-6}$	0.002*
NLR	0.644	0.560–0.728	57.1	69.5	11.806	0.001*
PLR	0.582	0.495–0.668	60.7	56.1	215.878	0.069
MLR	0.654	0.571–0.737	65.5	63.4	0.619	0.001*
CRP	0.610	0.523–0.696	36.9	89.0	1.500 mg/dL	0.015*
WBC	0.612	0.527–0.698	51.2	73.2	14.150 $\times10^{9}$/L	0.012*
Platelet	0.461	0.372–0.549	17.9	86.6	303.000$\times10^{9}$/L	0.381
Neutrophil	0.610	0.524–0.696	53.6	70.7	11.815$\times10^{9}$/L	0.014*
Lymphocyte	0.394	0.308–0.480	7.1	95.1	2.310$\times10^{9}$/L	0.018*
Monocyte	0.565	0.478–0.653	36.9	75.6	0.935$\times10^{9}$/L	0.146

mRS modified Rankin Scale, *AUC* area under the curve, *CI* confidence interval, *CLR* C-reactive protein to lymphocyte ratio, *NLR* neutrophil to lymphocyte ratio, *PLR* platelet to lymphocyte ratio, *MLR* monocyte to lymphocyte ratio, *CRP* C-reactive protein, *WBC* white blood cell, *$p < 0.05$ are considered significant.

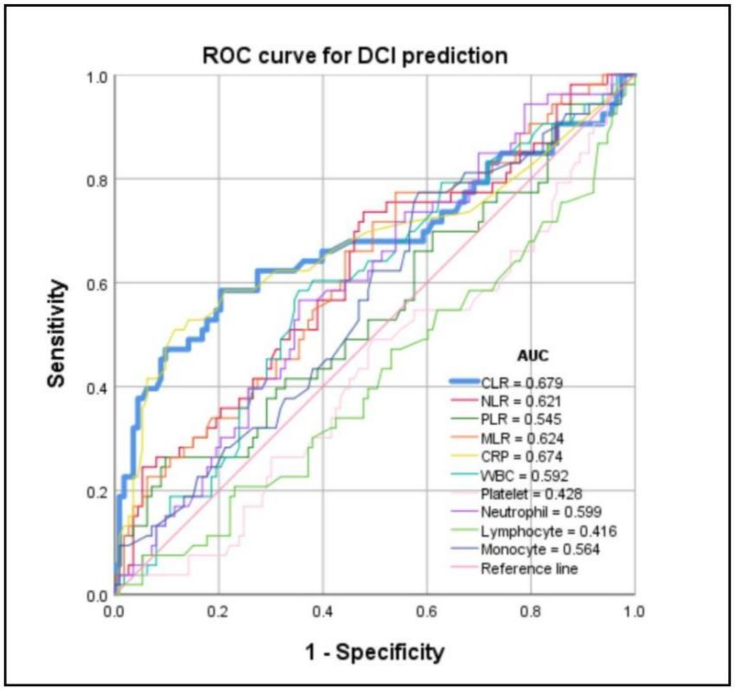

Supplemental Figure S2 Predictive value of each admission serum biomarkers for delayed cerebral ischemia (DCI) using receiver operating characteristics (ROC) curve.

Supplemental Table S8 Comparison of predictive value of different admission serum biomarkers associated with DCI.

Predictive biomarkers	AUC	95% CI	Sensitivity (%)	Specificity (%)	Cut-off value	*p* value
CLR	0.679	0.581–0.777	58.5	79.6	1.012 mg×10^{-6}	<0.001*
NLR	0.621	0.528–0.714	73.6	52.2	9.721	0.012*
PLR	0.545	0.448–0.642	26.4	90.3	398.444	0.350
MLR	0.624	0.533–0.715	77.4	46.0	0.562	0.010*
CRP	0.674	0.575–0.774	50.9	88.5	1.500 mg/dL	<0.001*
WBC	0.592	0.500–0.684	58.5	64.6	13.850×10^9/L	0.056
Platelet	0.428	0.335–0.522	98.1	5.3	131.500×10^9/L	0.138
Neutrophil	0.599	0.508–0.689	56.6	64.6	11.720×10^9/L	0.041*
Lymphocyte	0.416	0.322–0.511	7.5	94.7	2.310×10^9/L	0.083
Monocyte	0.564	0.471–0.658	77.4	41.6	0.605×10^9/L	0.181

DCI delayed cerebral ischemia, *AUC* area under the curve, *CI* confidence interval, *CLR* C-reactive protein to lymphocyte ratio, *NLR* neutrophil to lymphocyte ratio, *PLR* platelet to lymphocyte ratio, *MLR* monocyte to lymphocyte ratio, *CRP* C-reactive protein, *WBC* white blood cell, *$p < 0.05$ are considered significant.

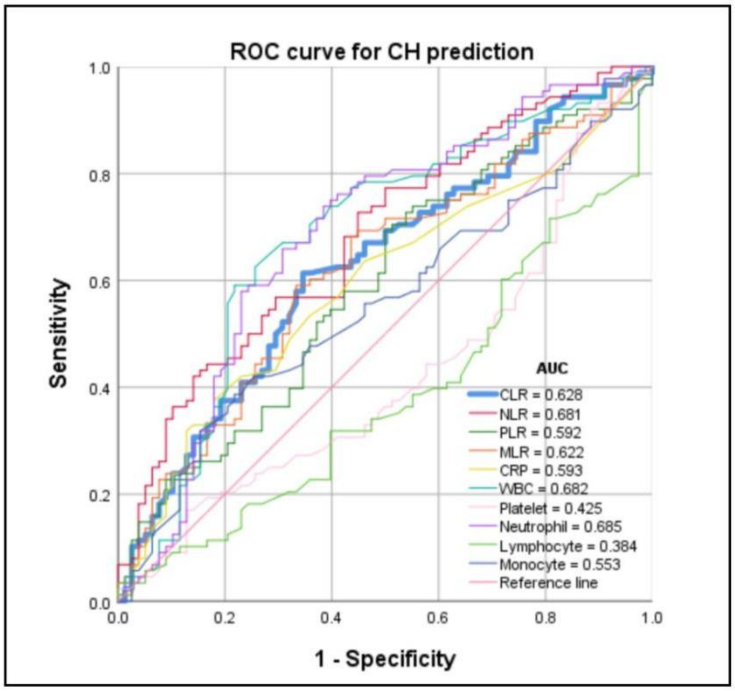

Supplemental Figure S3 Predictive value of each admission serum biomarkers for chronic hydrocephalus (CH) using receiver operating characteristics (ROC) curve.

Supplemental Table S9 Comparison of predictive value of different admission serum biomarkers associated with CH.

Predictive biomarkers	AUC	95% CI	Sensitivity (%)	Specificity (%)	Cut-off value	*p* value
CLR	0.628	0.543–0.713	61.4	65.4	0.510 mg×10^{-6}	0.005*
NLR	0.681	0.601–0.762	42.0	85.9	15.845	<0.001*
PLR	0.592	0.505–0.679	69.3	50.0	189.583	0.041*
MLR	0.622	0.537–0.707	59.1	66.7	0.658	0.007*
CRP	0.593	0.506–0.680	37.5	82.1	1.150 mg/dL	0.039*
WBC	0.682	0.598–0.766	59.1	78.2	13.950 ×10^9/L	<0.001*
Platelet	0.425	0.337–0.513	17.0	87.2	306.500×10^9/L	0.096
Neutrophil	0.685	0.602–0.768	73.9	61.5	10.435×10^9/L	<0.001*
Lymphocyte	0.384	0.299–0.469	96.6	0	3.455×10^9/L	0.010*
Monocyte	0.553	0.465–0.641	38.6	78.2	0.935×10^9/L	0.241

CH chronic hydrocephalus, *AUC* area under the curve, *CI* confidence interval, *CLR* C-reactive protein to lymphocyte ratio, *NLR* neutrophil to lymphocyte ratio, *PLR* platelet to lymphocyte ratio, *MLR* monocyte to lymphocyte ratio, *CRP* C-reactive protein, *WBC* white blood cell, *$p < 0.05$ are considered significant.
